# The choice of new treatments in autoimmune hemolytic anemia: how to pick from the basket?

**DOI:** 10.3389/fimmu.2023.1180509

**Published:** 2023-04-24

**Authors:** Sigbjørn Berentsen, Bruno Fattizzo, Wilma Barcellini

**Affiliations:** ^1^ Department of Research and Innovation, Haugesund Hospital, Helse Fonna Hospital Trust, Haugesund, Norway; ^2^ Fondazione IRCCS Ca’ Granda Ospedale Maggiore Policlinico, and Department of Oncology and Hemato-Oncology, University of Milan, Milan, Italy; ^3^ Fondazione IRCCS Ca’ Granda Ospedale Maggiore Policlinico, Milan, Italy

**Keywords:** clinical trials, complement inhibitors, cold agglutinin disease, corticosteroids, immune suppression, rituximab, therapy, autoimmune hemolytic anemia

## Abstract

Autoimmune hemolytic anemia (AIHA) is defined by increased erythrocyte turnover mediated by autoimmune mechanisms. While corticosteroids remain first-line therapy in most cases of warm-antibody AIHA, cold agglutinin disease is treated by targeting the underlying clonal B-cell proliferation or the classical complement activation pathway. Several new established or investigational drugs and treatment regimens have appeared during the last 1-2 decades, resulting in an improvement of therapy options but also raising challenges on how to select the best treatment in individual patients. In severe warm-antibody AIHA, there is evidence for the upfront addition of rituximab to prednisolone in the first line. Novel agents targeting B-cells, extravascular hemolysis, or removing IgG will offer further options in the acute and relapsed/refractory settings. In cold agglutinin disease, the development of complement inhibitors and B-cell targeting agents makes it possible to individualize therapy, based on the disease profile and patient characteristics. For most AIHAs, the optimal treatment remains to be found, and there is still a need for more evidence-based therapies. Therefore, prospective clinical trials should be encouraged.

## Introduction

We define autoimmune hemolytic anemia (AIHA) as anemia due to increased turnover of erythrocytes, caused by autoimmune mechanisms (1–3). The immune pathogenesis is usually mediated by autoantibodies against erythrocyte surface antigens, but the monocyte-macrophage system and T-lymphocytes are also involved (4, 5). It should be emphasized that AIHA is a collective term for several diseases (**Table 1**), classified according to findings by the monospecific direct antiglobulin test (DAT), the autoantibody class, the temperature optimum of the antigen-antibody reaction, and the absence or presence of an underlying or associated disease (6–8). The choice of optimal therapy differs between the AIHAs; therefore, exact diagnosis of the subtype is required to select the appropriate treatment (2).

**Table 1:** Autoimmune hemolytic anemias.

Warm-antibody autoimmune hemolytic anemia (wAIHA)	Cold-antibody autoimmune hemolytic anemia (cAIHA)	Atypical autoimmune hemolytic anemia
Primary wAIHA Secondary wAIHA Drug-induced AIHA	Cold agglutinin disease (CAD) Secondary cold agglutinin syndrome (CAS) Paroxysmal cold hemoglobinuria (PCH)	Mixed warm and cold AIHA DAT-negative AIHA

The first known case of what was likely an AIHA was described in the 13^th^ century by the Byzantine court physician Johannis Actuarius, who observed black urine in a patient after exposure to cold temperatures (9). Probably, his patient suffered from paroxysmal cold hemoglobinuria (PCH). Karl Landsteiner discovered cold agglutinins in 1903 (10). It was not until 1938 that Dameshek & Schwartz draw a distinction between congenital and acquired hemolytic anemia and, two years later, postulated an immune mechanism based on the finding of *hemolysin* in patient sera (11). Until the turn of the millennium, treatment was mostly based on theoretical considerations, clinical experience, and expert opinion, but during the last two decades, several prospective studies have been conducted (1, 12–14). Currently, several new therapies have appeared and quite a few clinical trials are running (15, 16). Furthermore, not only the treatment options but also the clinical landscape of AIHA have changed over time (17, 18). The complexity of the disease group and the growing number of new treatment options underscore the need for a thorough diagnostic workup to provide a basis for selecting “the right therapy to the right patient” (17, 19, 20).

This review will first address the different types of AIHA and then discuss the optimal selection of therapy based on AIHA type, disease features, and patient characteristics, with particular emphasis on novel therapies.

## The AIHA landscape and established therapies

### Warm-antibody AIHA

Approximately 70% of AIHA cases are warm antibody-mediated (wAIHA). In wAIHA, the autoantibodies are polyclonal, mostly directed at erythrocyte antigens of the Rh system, and have a temperature optimum for antigen binding at 37°C. They are mostly of the immunoglobulin G (IgG) class, but occasionally, IgA or warm-reactive IgM can be involved, alone or in combination with IgG (1, 8, 21, 22). Of the IgG subclasses, involvement of IgG1 is predominant, either alone or in combination with other subclasses (23–25). IgG1 antibodies have complement-activating properties and also high affinity for the neonatal Fc-receptor (FcRn) which affects IgG half-life (26, 27), both of which are thought to contribute to the severity and persistence of hemolysis.

Phagocytosis of immunoglobulin-opsonized erythrocytes by macrophages of the mononuclear phagocytic system, to a large extent in the spleen, is an important mechanism of extravascular hemolysis (28). Complement-mediated red blood cell destruction is involved in about 50% of the patients. The polyspecific (“simple”) DAT is used to confirm autoimmune pathogenesis by detecting antigen-bound immunoglobulin and/or complement on the erythrocyte surface (29, 30). The specific immunoglobulin class(es) and the occurrence of complement on the erythrocytes can be identified by the monospecific (“extended”) DAT, in which diagnostic antibodies specific for IgG, IgM, IgA, complement protein fragment 3c (C3c), and C3d are used as reagents (1, 2, 30, 31).

WAIHA occurs as a primary disease in slightly less than 50% of the cases and secondary to other disorders in the remaining cases (7, 8, 32, 33). The underlying or associated diseases in secondary cases are listed in **Table 2**. Occasionally, more than one associated disease is present. Evans’ syndrome was originally defined as AIHA with thrombocytopenia (34); now usually defined as the simultaneous or sequential combination of at least two autoimmune cytopenias, most often AIHA with immune thrombocytopenia (35–37). Infection-induced exacerbation of a preexisting primary wAIHA is not regarded as secondary.

**Table 2:** Underlying or associated conditions in secondary warm AIHA.

Lymphoproliferative disorders	B-cell lymphoma (Chronic lymphocytic leukemia, non-Hodgkin B-cell lymphomas) T-cell lymphoma (T-LGL leukemia, angioimmunoblastic T-cell lymphoma) Hodgkin lymphoma Castleman disease
Other hematologic disorders	Myeloid neoplasms (Myelodysplastic syndrome, myelofibrosis) An associated immune cytopenia, usually ITP (Evans’syndrome)
Solid tumors	Ovarian dermoid cyst or carcinoma Thymoma
Non-hematologic autoimmune and inflammatory diseases	Rheumatologic diseases (SLE, Rheumatoid arthritis, Sjögren syndrome) Antiphospholipid syndrome Autoimmune hepatitis Ulcerative colitis Sarcoidosis Eosinophilic fasciitis
Primary immunodeficiencies	Common variable immunodeficiency Autoimmune lymphoproliferative syndrome
Infections	Viruses (EBV, hepatitis, CMV, HIV, SARS-CoV-2) Bacteria (Tuberculosis, brucellosis) Protozoa (Babesiosis)
Transplantations	Allogenic bone marrow transplantation Organ transplantation (Liver, small bowel)

Some ultra-rare conditions have been omitted. CMV, cytomegalovirus; EBV, Epstein-Barr virus; HIV, human immunodeficiency virus; ITP, immune thrombocytopenia; LGL, large granular lymphocyte; SARS-CoV, severe adult respiratory syndrome corona virus; SLE, systemic lupus erythematosus.

**Figure 1:**
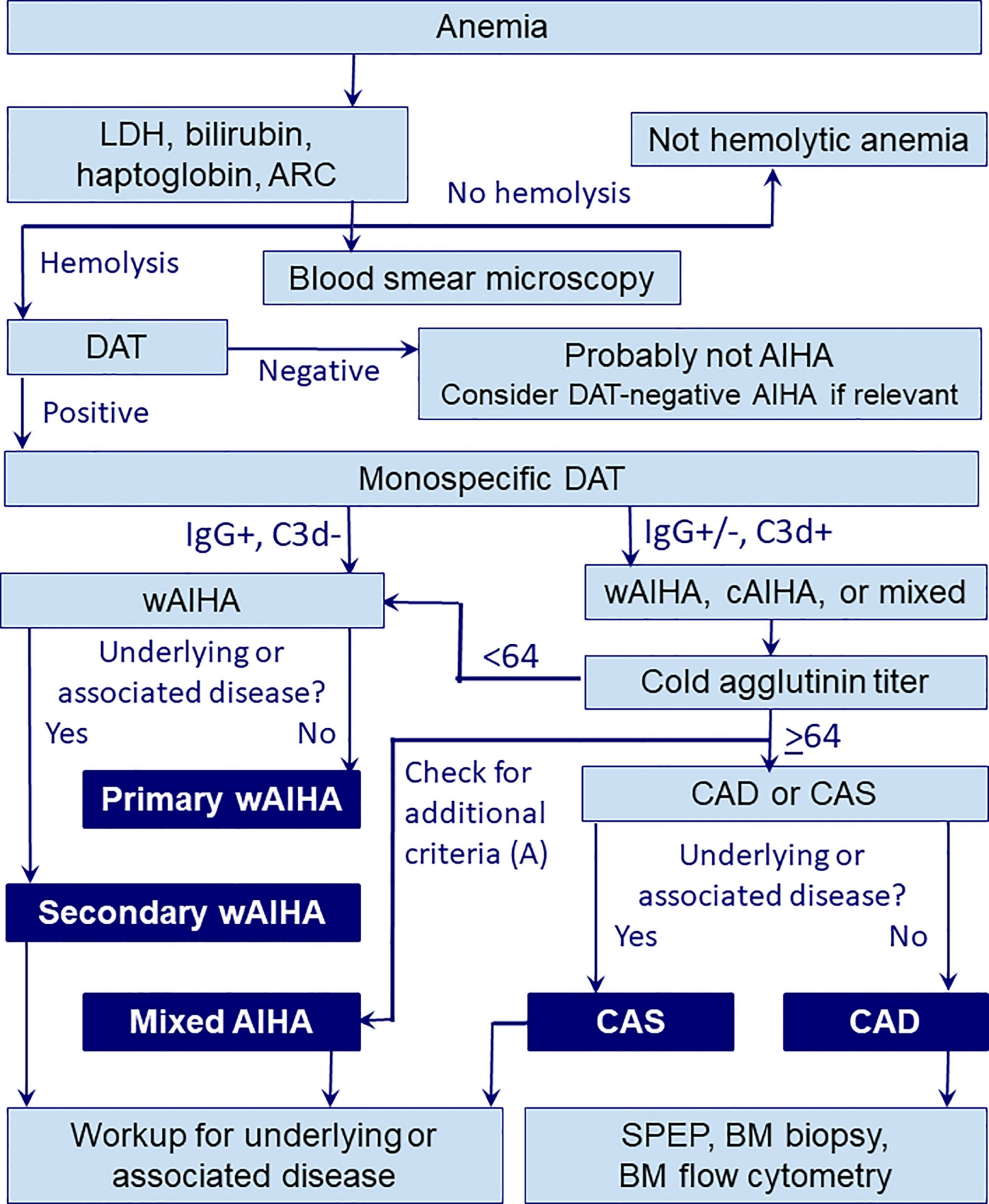
Diagnostic workup in AIHA. A, additional criteria for mixed AIHA (DAT strongly positive for both IgG and C3d, cold agglutinin titer ≥64, indirect antiglobulin test positive for IgG at 37°C); ARC, absolute reticulocyte count; BM, bone marrow; CAD, cold agglutinin disease; cAIHA, cold-antibody autoimmune hemolytic anemia; CAS, cold agglutinin syndrome; DAT, direct antiglobulin test; LDH, lactate dehydrogenase; SPEP, serum protein electrophoresis; wAIHA, warm-antibody autoimmune hemolytic anemia.

**Figure 1** shows an algorithm for diagnostic workup of AIHA including wAIHA. It should be noted that although the absolute reticulocyte count is usually elevated because of increased erythrocyte turnover and bone marrow compensation, some patients have normal counts or even reticulocytopenia (38–40). This is probably explained by autoantibody activity against erythroid precursors (40), but T-cell mediated suppression or inflammatory mechanisms may also contribute. WAIHA carries an increased risk of thrombosis, which, in addition to a risk of infection and other treatment complications, probably contributes to increased mortality (41–48). In most cases, wAIHA is a chronic relapsing rather than a chronic disease. Usually, patients are symptomatic at presentation and require treatment.

Prednisolone (or prednisone), administered at high initial doses (100 mg fixed dose or 1-1.5 mg/kg body weight daily), then slowly tapered after 2-3 weeks and discontinued after about 4-6 months, remains first-line therapy in wAIHA and yields responses in approximately 80% of the patients (1, 43, 49–51). Other oral corticosteroids have not shown higher efficacy (1, 52).

Patients who fail to respond to first-line therapy should be considered for a new diagnostic workup to reveal any overlooked underlying or concomitant disease that might be subject to specific therapy, such as associated autoimmune conditions, lymphoproliferative disorders, other neoplasms, or non-immune hemolytic anemias (1, 8, 53). In addition to a thorough clinical evaluation and review of the records and medication history, this assessment should include extensive autoantibody panels, serum protein electrophoresis, bone marrow examination, and exclusion of paroxysmal nocturnal hemoglobinuria and congenital hemolytic anemias (54).

Rituximab at a conventional (375 mg/m^2^ at 1-week interval for 4 weeks) or low dose (100 mg fixed dose at the same schedule) is currently recommended in the second line in primary wAIHA (1, 51, 55, 56). However, there are no published data to support repeated use of rituximab in wAIHA patients who have received this monoclonal antibody and subsequently relapsed. Third-line therapies include unspecific immune suppressants, such as azathioprine, cyclosporine, mycophenolate, and others (1, 57). Splenectomy, which was previously often recommended in the second line, is now considered an option in the third or subsequent lines (1, 2, 14, 58).

With some exceptions, recommended first-line treatment in secondary wAIHA is generally as for primary wAIHA (1, 2, 51). Treating the underlying or associated disease is indicated if this disease requires treatment by itself or if first-line therapy of the AIHA has failed.

### Cold-antibody AIHA

#### Cold agglutinin disease

Cold agglutinin disease (CAD) accounts for 20-30% of AIHA cases (6, 59). The autoantibodies responsible for hemolysis in CAD are termed cold agglutinins, referring to their ability to agglutinate erythrocytes at temperatures below 37°C; such temperatures are normally found in the acral parts of the body (60). Cold agglutinins in CAD are monoclonal, usually of the IgM class with κ light chain restriction (61, 62). The cold agglutinin-producing pathogenetic process is a clonal B-cell lymphoproliferative disorder of the bone marrow, now recognized as a distinct entity by the World Health Organization classification of hematolymphoid neoplasms, but not considered to be a malignant lymphoma (63–65).

Binding of cold agglutinin to its cell surface antigen results in agglutination of erythrocytes, leading to complement-mediated hemolysis and, often, circulatory symptoms such as acrocyanosis and Raynaud-like phenomena (16, 61). Of note, the agglutination and circulatory symptoms are not complement-mediated. Complement is activated *via the* classical pathway, triggered by fixation of the C1qrs complex to antigen-bound IgM. Activated C1s cleaves C4 and C2, resulting in the formation of C3 convertase, coating of the erythrocytes with C3b, and phagocytosis of opsonized erythrocytes by macrophages (extravascular hemolysis), mainly in the liver (66–69). Especially in severely affected patients or acute exacerbations, split products may also combine to form C5 convertase on the cell surface, resulting in formation of the C5b-9 complex and intravascular hemolysis. The soluble split products, C3a and C5a, have proinflammatory properties and are thought to result in, or contribute to, fatigue in many patients with CAD (16, 70, 71).

**Figure 2:**
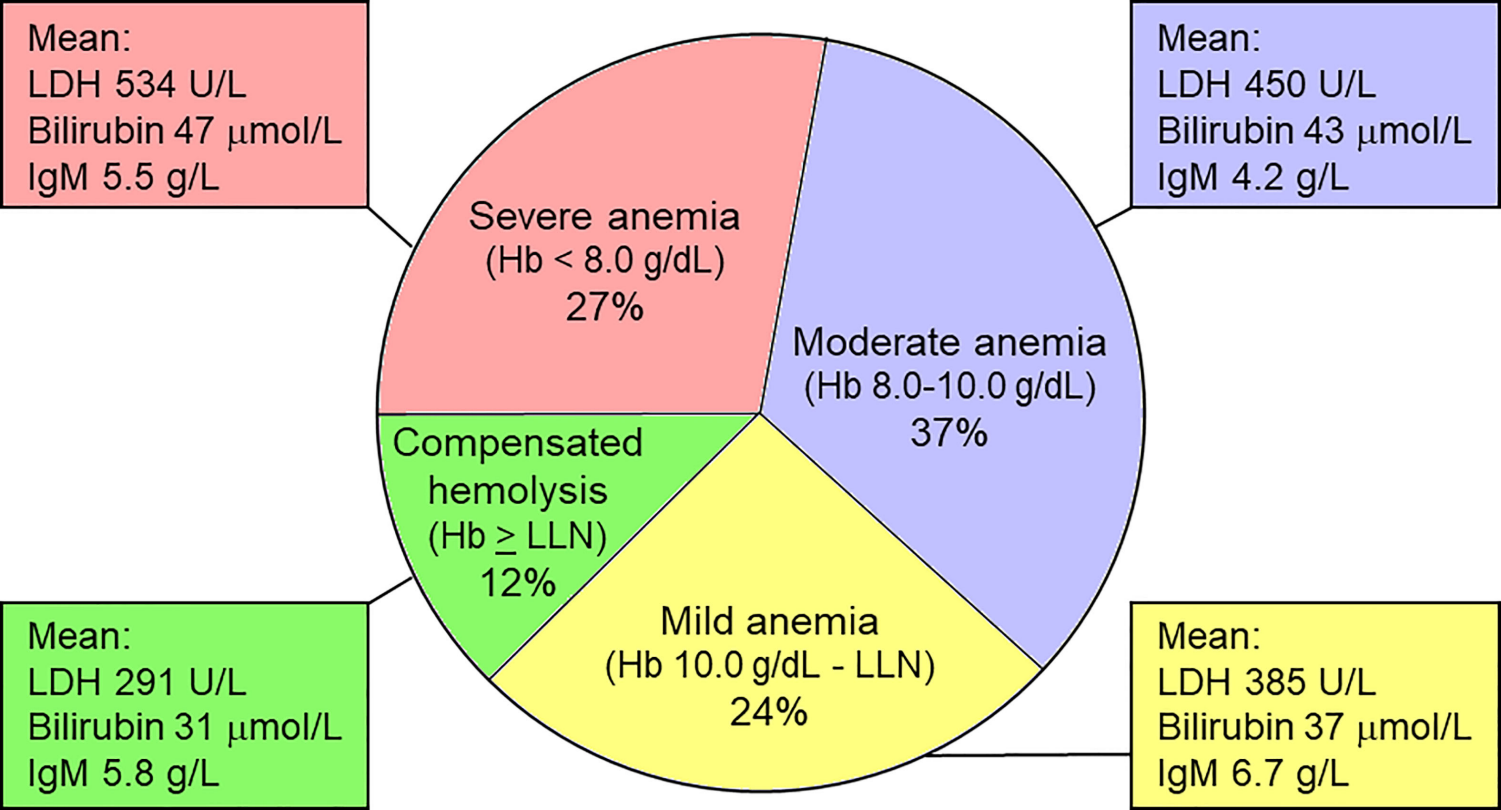
Severity of anemia in CAD. Hemoglobin levels correlate negatively with parameters of hemolysis, but do not correlate with IgM levels. LDH, lactate dehydrogense; LLN, lower limit of normal. Based on data from Berentsen et al. (61). Figure first published in *Front Immunol* 2020 by Berentsen (71), reused under a Creative Commons CC-BY license 4.0 (https://creativecommons.org/licenses/by/4.0/). ^©^ S. Berentsen 2020.

The severity of anemia in CAD is shown in **Figure 2** (61, 71). In a multinational, observational study, 146 patients (69.5% of 210 patients with available relevant data) had hemolytic anemia with no or mild peripheral circulatory symptoms, 44 (21%) had hemolytic anemia with circulatory symptoms interfering with daily living, while 20 (9.5%) had circulatory symptoms with compensated hemolysis (61). Patients have an increased risk of thrombosis (61, 72, 73).

Patients with no or mild symptoms have not been shown to benefit from being treated. Older literature, however, has probably underestimated the symptom burden and need for therapy (61, 72, 74). Taking the increasing number of effective therapies into account, we consider symptomatic anemia, marked fatigue, or bothersome circulatory symptoms as indications for treatment (2, 20, 75). CAD should not be treated with corticosteroids, unspecific immune suppression, or splenectomy (1, 2, 20, 61, 76). Treatments directed at the pathogenic B-cell clone or the classical complement pathway have shown efficacy (61, 70, 77–85), as listed in **Table 3**. Rituximab at the dose of 375 mg/m^2^/week for 4 weeks is the most widely used first-line therapy, although response rates are modest and response duration is relatively short (78, 79). Addition of bendamustine highly improves response rates and duration but is also associated with some toxicity (61, 81).

**Table 3:** Published studies of therapy in CAD.

Target	Treatment	Study (Reference)	Study design	ORR^1^ (%)	CR^2^ rate (%)	Median response duration (months)	Toxicity
B-cell directed theapies	Rituximab monotherapy	Berentsen et al., 2004 (78) Schöllkopf et al., 2006 (79)	Prospective, non-randomized	45-55	<5	6.5-11	Low
	Rituximab plus fludarabine	Berentsen et al., 2010 (80)	Prospective, non-randomized	76	21	>66	Significant
	Rituximab plus bendamustine	Berentsen et al., 2017 (81) Berentsen at al. 2020 (61)	Prospective, non-randomized	78	53	>88	Moderate, manageable
	Bortezomib monotherapy	Rossi et al., 2018 (82)	Prospective, non-randomized	32	16	>16	Low
	Ibrutinib monotherapy	Jalink et al., 2021 (83)	Retrospective	100	NR^1^	ND^1^	Low
Complement-directed therapies	Sutimlimab	Röth et al. (CARDINAL study) 2021 (70)	Prospective, non-randomized	>73^3^	NR^1^	>24	Low
		Röth et al. (CADENZA study) 2021 (77)	Prospective, randomized				
	Pegcetacoplan	Grossi et al., 2018	Part of prospective phase 2 study	ND/high^3^	NR^1^	ND^1^	Low

^1^ORR, overall response rate; ND, not determined; NR, not relevant.

^2^CR, complete response. Criteria for CR included eradication of detectable bone marrow lymphoproliferative disorder.

^3^ORR was not an endpoint of this study. Estimated ORR is based on data from the original publication.

#### Secondary cold agglutinin syndrome

In secondary cold agglutinin syndrome (CAS), cold agglutinin-mediated AIHA is caused by, or associated with, another clinical disease, such as infection with *Mycoplasma pneumoniae*, Epstein-Barr virus, cytomegalovirus, SARS-CoV-2, or other viruses, or a malignant disease, most often an overt B-cell lymphoma (86–92). There is no evidence-based therapy except for treating the underlying disease, if possible. Upstream complement inhibition has a strong theoretical rationale as a temporary measure until treatment of the underlying disorder takes effect, but the benefit remains unproven except for casuistic observations (84, 93).

#### Paroxysmal cold hemoglobinuria

PCH is an AIHA mediated by a polyclonal, biphasic antibody (Donath-Landsteiner antibody), usually an IgG (18, 94). Classical and terminal complement activation is strong and hemolysis is mainly intravascular. The term biphasic implies that the antigen-antibody reaction and fixation of the early complement components occur at temperatures below 37°C, whereas further complement activation takes place after rewarming to central body temperature (18, 95). Today, most cases of this rare condition occur as an acute, transient hemolytic anemia following a viral or other febrile infection in children (18, 96). The chronic paroxysmal (adult) form of PCH has become extremely rare and can occur in tertiary syphilis, hematologic malignancies, or without any identifiable cause.

### Atypical AIHA

*Mixed warm and cold-antibody AIHA* is characterized by a monospecific DAT strongly positive for both IgG and C3d, high-titer cold agglutinins, and the concomitant presence of IgG warm-antibodies in serum (8, 97). Many patients have severe anemia and receive multiple lines of therapy before responding.

*DAT-negative AIHA.* In 3-10% of patients with AIHA, the polyspecific DAT is negative, mostly due to warm-autoantibody opsonization below the detection level. The diagnosis of DAT-negative AIHA remains difficult and requires extensive exclusion of differential diagnoses (non-immune causes of hemolysis) (1, 19, 54). More sensitive methods for detecting immunoglobulin on the erythrocyte surface may be helpful, but have lower specificity. A monospecific DAT for IgA is also mandatory, despite the negative polyspecific test (2, 54).

## How to select therapy in warm AIHA

### When to add rituximab in the first line?

As stated above, prednisolone (or prednisone), monotherapy is recommended in the first line in most patients with wAIHA. However, the low rate of long-sustained remission after tapering and discontinuation (30-40% after 1 year) is a concern, particularly in severely affected patients (98–100). In clinical practice, patients face a risk of being maintained on inappropriately high doses of steroids for years (14).Two prospective, randomized trials evaluated the upfront addition of rituximab (either 4 infusions of 375 mg/m^2^ at one-week interval or 2 infusions of 1000 mg fixed dose at two-week interval) (99, 100). The results were nearly identical, showing twice the rate of long-term responses in patients who had been given the combination upfront as compared to those treated with prednisolone only. Therefore, the international consensus group has recommended that severely anemic patients (defined as hemoglobin [Hb] < 8 g/dL) should be considered for rituximab plus prednisolone combination therapy in the first line (1). In this context, atypical AIHA (IgA-mediated, DAT-negative, or mixed) and Evans’ syndrome are also regarded as severe (1, 2, 22, 54, 101).

### When to use erythropoiesis-stimulating agents?

The use of erythropoiesis-stimulating agents (ESA; erythropoietin [EPO] or its analogs) in AIHA has been studied in two retrospective series (102, 103). In the largest and most recent of these, 51 patients with warm or cold-antibody AIHA and reduced EPO levels or inadequate EPO response had been treated with an ESA, starting at median 24 months after diagnosis (103). Fifty-five percent of the patients responded within 15 days and more than 70% responded within 3 months; most responses seemed to be sustained on continued therapy, and the median increase in Hb was 2.1 g/dL. An attempt of ESA treatment will be worthwhile in patients with inadequate reticulocytosis and/or inadequate endogenous EPO levels at disease onset as well as after failing on corticosteroids, rituximab, and an immunosuppressive agent. For patients in whom corticosteroid maintenance is inevitable, an ESA may be successful as a steroid-sparing drug.

### When to use intravenous immunoglobulin in wAIHA?

Administration of intravenous immunoglobulin (IVIG) at a total dose of 2 g/kg body weight (0.4 g/kg day for 5 days or 1 g/kg for 2 days) is supposed to inhibit extravascular hemolysis by saturating the reticuloendothelial system and Fc receptors (FcR) (104, 105). Saturating the FcRn will also promote clearance of the autoantibody (106). IVIG may be considered in hemolytic crisis and may be particularly useful in septic patients and those with an underlying autoimmune disease or primary immune deficiency.

### When to recommend splenectomy?

Although splenectomy is far from being a new treatment for wAIHA, its position in the therapeutic armamentarium has changed. The response rates are high; probably 70-80% (8, 107), but long-term data are sparser (108). Disadvantages are a risk of early or late bacterial infection, a further increased risk of thrombosis, and the irreversibility of the procedure (109, 110). Therefore, we only recommend splenectomy in the third or subsequent lines. Although several experts will postpone splenectomy until at least one unspecific immunosuppressant has been tried, patient preferences should be taken into account (14, 111). However, considering the difficulties in treating some wAIHA patients and the relatively high efficacy of removing the spleen, this option should not be withheld in drug-refractory cases (1, 14). Patients should be vaccinated against encapsulated bacteria according to national recommendations or the Advisory Committee on Immunization Practices (ACIP) guidelines (112).

### When to use investigational therapies in wAIHA?

#### Clinical trials

The ideal therapy for wAIHA should be low-toxic, easy to administer, should yield high response rates and durable responses, and should substantially reduce the high risk of relapses. Such a therapy does currently not exist. With this in mind, more prospective clinical trials are needed (12, 14, 15). Patients requiring therapy in any line may be considered for a prospective trial if eligible, provided randomization to the control arm (if relevant) is considered ethically justifiable.

#### Inhibitors of the neonatal Fc receptor

FcRn is required for physiological recirculation of IgG, which explains the long normal biological half-life of IgG1, IgG2, and IgG4 at approximately 23 days (27, 106). Blockade of this receptor, therefore, results in marked shortening of IgG half-life and lowering of IgG levels to about 10% of normal without inhibiting the production (113). Nipocalimab (M281), rozanolixizumab (UCB7665), and orilanolimab (SYNT001) are FcRn-targeting monoclonal antibodies (114, 115), and the safety and efficacy of nipocalimab is being studied in a prospective trial in wAIHA (ClinicalTrials.gov, NCT04119050). Available clinical data do not permit suggestions for its future role in treatment. However, the very quick and reversible pharmacodynamics observed in preclinical trials may suggest their use in the acute setting, similarly to IVIG employment.

#### Fostamatinib

Inhibitors of cellular mediators of phagocytosis are currently being investigated for AIHA treatment. One of these drugs, fostamatinib, a splenic tyrosine kinase (syk) inhibitor, was found to increase Hb levels to ≥10 g/dL or by 2 g/dL or more in 11 (46%) of 24 patients with wAIHA in a phase 2 trial (116). Participants had failed at least one previous treatment. Forty-two per cent of the patients experienced diarrhea; and fatigue, hypertension, dizziness, and insomnia were also frequent adverse events. The drug is currently approved in the US and Europe for treatment of immune thrombocytopenia. A phase 3 trial in wAIHA has finished inclusion (117), and an open-label extension study is also being performed (ClinicalTrials.gov, NCT03764618; NCT04138927). While still investigational, fostamatinib may turn out to be an option in the third line.

#### Other inhibitors of signal transducers

Rilzabrutinib, a reversible, covalent Bruton tyrosine kinase (BTK) inhibitor (118), is currently being studied in a phase 2 trial in wAIHA (ClinicalTrials.gov, NCT05002777). This drug also inhibits phagocytosis *via* interaction with the syk pathway. Parsaclisib, a phosphatidylinositol 3-kinase δ inhibitor, is a candidate drug in wAIHA as well as CAD (119). Preliminary phase 2 results in relapsed or refractory wAIHA show a 64% response rate, although with some toxicities including diarrhea, cytomegalovirus reactivation, and psoriasis (120). A randomized, controlled phase 3 trial is ongoing (ClinicalTrials.gov, NCT05073458).

#### Plasma cell-directed therapies

Bortezomib, a potent and selective proteasome inhibitor extensively used for the treatment of multiple myeloma, has showed promising results in autoimmune disorders by targeting the antibody-producing cells, including long-lived plasma cells (121). Beneficial effect of bortezomib in refractory wAIHA has been described in several case reports (122). In a retrospective series of adults who received 6 cycles of bortezomib plus dexamethasone, 6 of 8 patients responded following a median of 2 (range, 1-4) cycles (123). A small, prospective study found response to a combination of bortezomib, low-dose rituximab, and dexamethasone in 6 of 7 patients with refractory wAIHA (124).

At present, the evidence for bortezomib in wAIHA is limited because evaluation of pooled case reports is likely to be influenced by selection and publication bias and because the two systematic studies are small. Despite these reservations, bortezomib-based therapy may accommodate a previously unmet need in refractory or relapsed patients in whom rituximab has failed or is contraindicated.

In a small case series, therapy with the CD38-targeting monoclonal antibody daratumumab was used as a last resort in 3 patients with life-threatening, refractory wAIHA after stem cell transplantation (125). Two patients enjoyed a complete and sustained response with only minor toxicity, while the third patient experienced a transient improvement for 8 months but then suffered a lethal relapse. A systematic study, for example a larger retrospective series, should be performed before the potential role of daratumumab in wAIHA can be determined. The interference of daratumumab with DAT and indirect antiglobulin testing should be observed (126). A phase 1 study of isatuximab, an anti-CD38 monoclonal antibody for subcutaneous administration in wAIHA, is ongoing (ClinicalTrials.gov, NCT04661033).

#### Sirolimus

Sirolimus, an immunosuppressive ingredient of *Streptomyces hygroscopicus*, has been found to prevent graft rejection following organ transplantations (127). Administration of this drug was followed by remission in 4 children with wAIHA; 2 of them with overlapping pure red cell aplasia (128). Beneficial effect has also been observed in children with Evans’ syndrome (129). A retrospective study of sirolimus therapy in 45 adults and children with relapsed or refractory autoimmune cytopenias, of whom 14 had primary wAIHA and 12 had Evans’ syndrome, reported favorable response and safety data (127). Sirolimus may find a future role in treatment of refractory wAIHA including Evans’ syndrome in children, possibly also in adults, but should still be considered experimental as prospective clinical studies are lacking. Frequent hematological, gastrointestinal, cardiac, and immunological adverse effects may be a concern. A prospective study would be welcome.

### What to do in emergencies?

Suggested emergency therapies in critically anemic wAIHA patients with no or slow response to prednisolone have been addressed elsewhere and include high-dose intravenous methylprednisolone, IVIG, plasma exchange, emergency splenectomy, and partial splenic embolization (1, 2, 51, 130, 131). While success has been documented in single cases, the evidence supporting each of these options is limited, and most of them are old and not within the scope of this review.

Regarding newer approaches in the emergency setting, prompt effect of complement inhibition with high, frequently repeated doses of plasma-derived C1-inhibitor was reported in a single case of severe, secondary IgM-mediated wAIHA with complement involvement (132). However, the preliminary results of a prospective trial seem disappointing (133). Using the specific C1s-targeting, monoclonal antibody sutimlimab in this context has a strong mechanistic rationale but has not been systematically studied. Still, based on theoretical considerations, case observations, and immediate effect on the classical complement pathway, C1 inhibition may be considered a low-toxic and potentially efficient option in life-threatening wAIHA with a positive DAT for C3 fragments (1, 132, 134). Furthermore, a phase 2 study of the C3 inhibitor pegcetacoplan showed some efficacy in wAIHA with C3-positive DAT (135).

Bortezomib-based combination regimens, addressed above, might also be used in emergencies based on the high response rates reported in small series and relatively low toxicity (123, 124). Administration of an ESA can be a useful supplement in a hemolytic crisis (1, 103). Potential future agents for the acute setting may also include the already mentioned FcRn inhibitors that may boost the clearance of the autoantibodies, thus limiting the hemolytic crisis (27, 106, 114).

**Figure 3:**
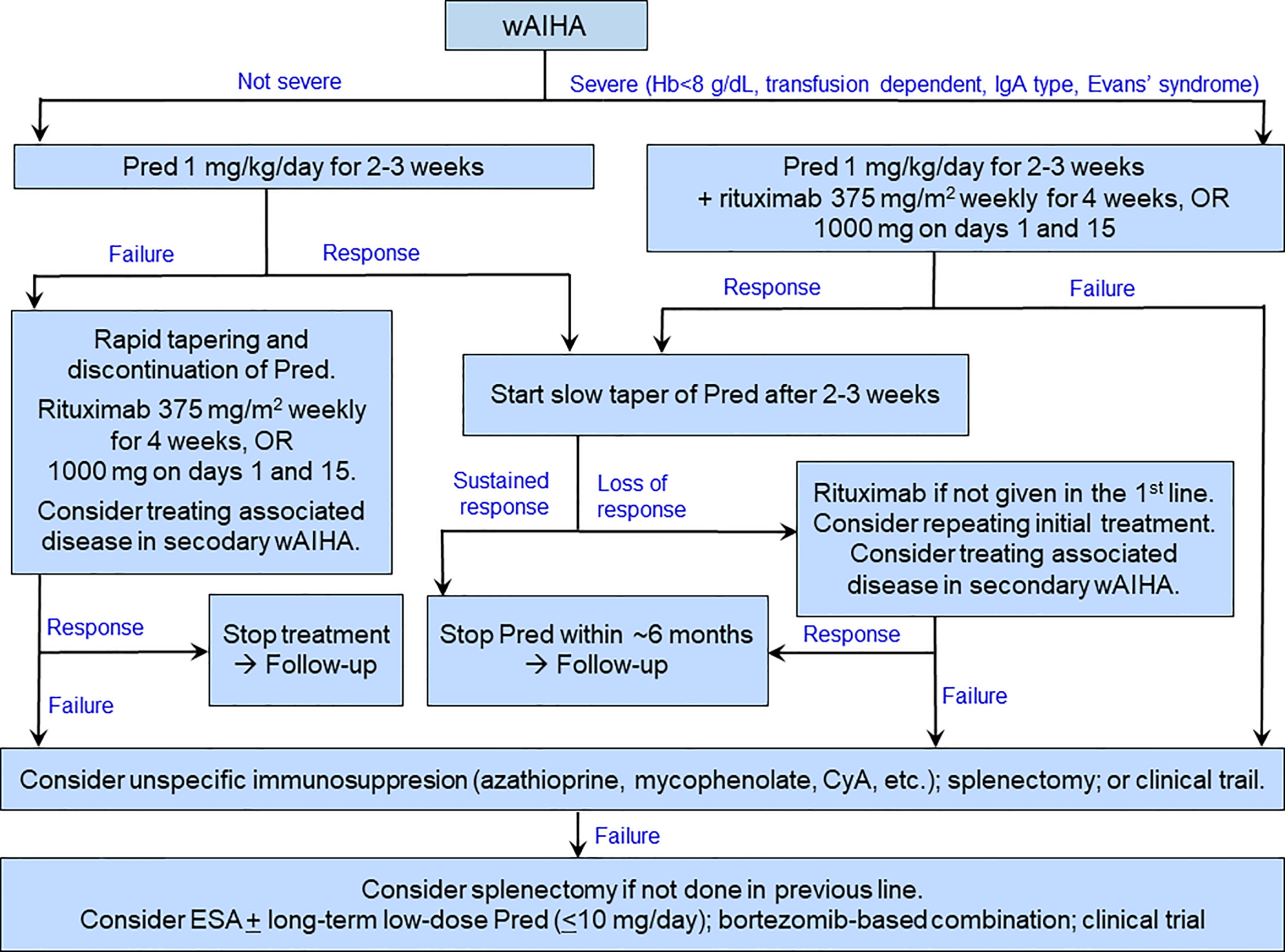
Suggested therapeutic algorithm in warm-antibody AIHA. ESA may be used as a supplement at any stage as well as in the last line. CyA, cyclosporine A; ESA, erythropoiesis-stimulating agent; Pred, prednisolone or prednisone.

**Figure 3** shows a therapeutic algorithm covering the most commonly encountered situations in wAIHA patients.

## How to select therapy in CAD

### B-cell or complement directed therapy?

**Figure 4:**
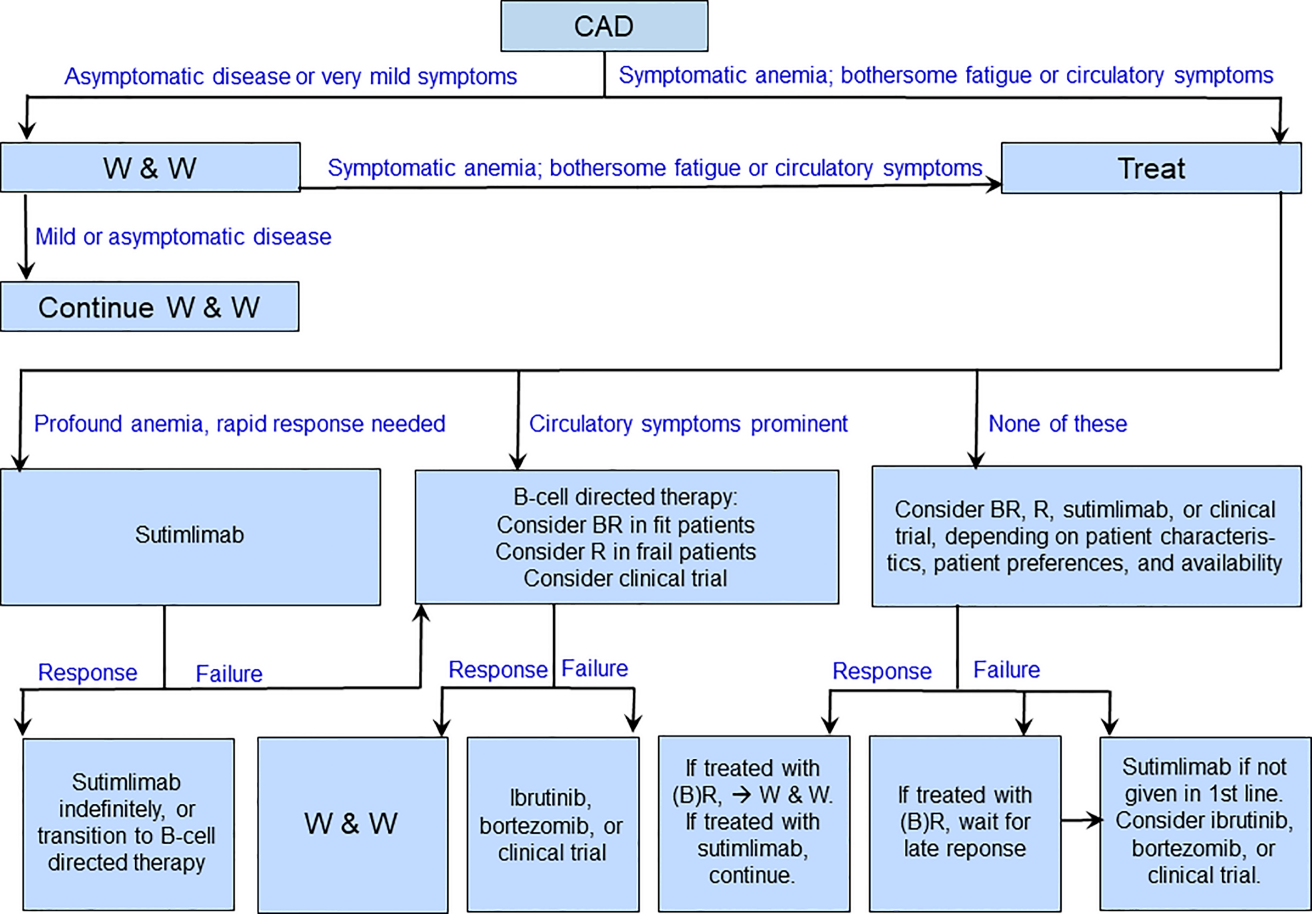
Suggested therapeutic algorithm in cold agglutinin disease. B, bendamustine; R, rituximab; W&W, watch and wait.

Although comparative trials of B-cell directed therapies *versus* complement inhibition have not been performed, some conclusions on preferences are justified based on the different profiles of these approaches (16, 136, 137), as indicated in **Table 4**. Advantages of the existing B-cell directed therapies are the time-limited treatment, the high rate of overall and complete responses and long response duration with bendamustine plus rituximab, the effect on circulatory symptoms as well as hemolytic anemia, and the relatively low toxicity of rituximab monotherapy. Drawbacks are the often-long time to response, the existing although usually manageable toxicity of bendamustine plus rituximab, and the relatively low response rate and short response duration with rituximab monotherapy (61, 78, 81). The most extensively studied complement inhibitor, sutimlimab, has obvious advantages in the very rapid onset of effect, the high response rate, and the low toxicity. Disadvantages of sutimlimab are the lack of effect on circulatory symptoms, the probable need for indefinite treatment duration, the biweekly intravenous infusions, and the very high costs (136, 138). **Figure 4** shows a suggested therapeutic algorithm.

**Table 4:** B-cell directed *versus* complement-directed therapies in CAD.

Therapy	Study (Reference)	Overall efficacy	Time to response	Effect on hemolysis	Effect on circulatory symptoms	Duration of therapy	Response duration	Toxicity	Costs
**Rituximab monotherapy**	Berentsen et al., 2004 (78); Schöllkopf et all. 2006 (79)	Modest	Sometimes long	Moderate	Moderate	Short	Short; therapy can be repeated	Low	Moderate
**Bendamustine plus rituximab**	Berentsen et al., 2017 (81); 2020 (61)	High	Sometimes long	High	High	Short	Long. Some patients may be cured.	Moderate	Moderate
**Sutimlimab**	Röth et al., 2021 (70); 2022 (77)	High	Short	High	None	Indefinite	Long (provided continued treatment)	Low	Very expensive

### How to treat critically anemic patients with CAD?

Acute exacerbations of CAD are often triggered by cold exposure, febrile infection, major trauma, or major surgery (61, 139, 140). In these situations, it is reasonable to treat the cause of exacerbation when relevant, transfuse the patient if required and, in many cases, wait several weeks for improvement of the hemolytic anemia. In CAD patients who present with profound anemia and need a rapid effect of therapy, first-line use of sutimlimab will often be successful, given the often-long time to response to B-cell directed therapies (70, 77, 136). After a stable response has been achieved, highly selected patients might be considered for transition to a time-limited, B-cell targeting therapy as an alternative to indefinite continuation of sutimlimab, depending on patient characteristics (fit or unfit), clinical disease features (marked circulatory symptoms or not), and patient preferences (136, 141). However, this “bridging” approach is currently not evidence-based.

### How to treat patients with prominent circulatory symptoms?

As explained above, peripheral circulatory symptoms are absent or mild in approximately two-thirds of patients with CAD, while about one-fifth have hemolytic anemia with circulatory symptoms interfering with daily living (61). Approximately one-tenth of patients have circulatory symptoms with compensated hemolysis, i.e. as the only or main clinical manifestation. Thus, circulatory symptoms may constitute, or substantially contribute to, the indication for treatment in up to 30% of the patients. As these symptoms are not complement-mediated, they are not expected to improve on complement-inhibiting therapy, which is in accordance with the findings in a clinical trial (142). Patients in this subgroup, therefore, should be treated with B-cell directed therapies, which have the potential to resolve circulatory symptoms as well as hemolysis (16, 78, 81).

### How to choose the best therapy in the “typical” CAD patient?

A high proportion of CAD patients requiring therapy have moderate anemia, often fatigue, and no or mild circulatory symptoms (**Figure 2**) (61). In such patients, current evidence does not allow any hard, general recommendation, and therapy should be individualized. In the first line, a B-cell directed approach will be appropriate in most of these patients (16, 137). Moderate to severe anemia in an otherwise fit patient will favor rituximab plus bendamustine, whereas mild clinical disease and/or unfit patient will favor rituximab monotherapy. In this setting, it is reasonable to consider sutimlimab as a second-line option, which may be used in the first line in patients who have contraindications to chemoimmunotherapy (136). Patient preferences should be taken into consideration. Second-line options, apart from sutimlimab, may include bortezomib (82) or inclusion in a clinical trial, while the use of rituximab plus fludarabine should be restricted to carefully selected patients (61, 80).

### When to consider inclusion in a clinical trial?

Even after the recent, major progress in the treatment of CAD, there are several unmet needs. The ideal therapy should be highly efficacious against hemolytic anemia and fatigue as well as circulatory symptoms, rapidly acting, low-toxic, and affordable worldwide. Since such therapies do not exist, clinical trials could be considered in any line of therapy provided randomization (if relevant) is deemed ethically justifiable.

Regarding future B-cell directed approaches, treatment with the BTK inhibitor ibrutinib has shown highly promising results in a retrospective series (83). In this study of 13 patients with CAD or CAS secondary to low-grade non-Hodgkin lymphoma, all patients responded; responses seemed sustained on continued medication, and the drug was generally well tolerated. This option should be considered investigational until completion of a prospective BTK inhibitor trial, which is warranted.

Complement-directed therapies should also be further explored. The C3 inhibitor pegcetacoplan (APL-2), a pegylated cyclic peptide designed for subcutaneous infusion, has yielded favorable response rates with low toxicity in a phase 2 study (85). Pegcetacoplan in CAD is currently being further investigated in a randomized, placebo-controlled phase 3 trial (ClinicalTrials.gov, NCT05096403). Other complement-targeting agents that may find a role in the treatment of CAD are ANX005, a C1q inhibitor (143, 144), BIVV020, which targets C1s (145) (ClinicalTrials.gov, NCT04269551), and ARGX-117, which inhibits C2 (146). Relevant clinical data have not been published for these three monoclonal antibodies, but prospective trials would be welcome.

### What to do in emergencies?

The traditional emergency therapy in CAD is plasma exchange (147–149). As 80% of IgM is located in the blood stream, the theoretical chance of success should be high. However, while favorable results have been confirmed in several cases, unsuccessful attempts may be underreported, and response rates have not been determined. The response duration is short, and concomitant initiation of pharmacological therapy is mandatory. For substitution, albumin should be used instead of plasma in order to avoid infusion of exogenous complement (51, 139).

Today, considering the very rapid effect of sutimlimab on hemolytic anemia in CAD, complement C1 inhibition may be preferred as an emergency therapy (1, 20, 70, 136). Even C5 inhibition with eculizumab may be helpful, as C5b-9 mediated, intravascular hemolysis can be prominent in critically hemolytic patients (1, 84, 150).

## How to select therapy in other AIHAs

### Secondary cold agglutinin syndrome

CAS caused by *Mycoplasma pneumoniae* or viruses will resolve spontaneously after elimination of the infection. In *Mycoplasma* infection, however, hemolytic anemia can occasionally be profound and prolonged, and the onset of the hemolytic complication typically occurs in the second week, when antibiotic therapy has often already been initiated or even completed (86, 88, 90). Transfusion is safe provided the same precautions are observed as in CAD (See below). Complement inhibition has a strong theoretical rationale, but clinical evidence is lacking except for a couple of case reports (93).

### Paroxysmal cold hemoglobinuria

In children with PCH, the use of corticosteroids has recently been questioned based on a literature study of all 230 published PCH cases, in which there was no difference in length of hospital stay between patients who had received corticosteroids and those who had not (18). In most patients, thermal protection and, if necessary, transfusion will be appropriate until spontaneous resolution occurs (151, 152). Terminal complement blockade has a strong mechanistic rationale; was followed by immediate resolution in a case report, and might be justified in selected cases as an attempt to relieve critical hemolytic anemia in pediatric PCH patients (96). In the extremely rare cases of adult PCH, there is no evidence-based therapy apart from thermal protection and treatment of the underlying disease, when identifiable.

### How to treat mixed AIHA?

Mixed warm and cold-antibody AIHA is often difficult to treat, and prospective trials specific for this rare AIHA have not been conducted. In our clinical experience, patients should receive corticosteroids at high doses along with rituximab in the first line (1, 2, 8, 153). If cold agglutinin-related clinical symptoms are prominent, it may be advisable to treat this subtype like CAD (2). In some cases, the same patient may display alternate clinical and laboratory features of wAIHA and CAD, requiring specific treatments. Splenectomy is discouraged.

## Which AIHA patients should receive erythrocyte transfusions?

Transfusion in AIHA requires specific precautions, depending on the temperature range of the autoantibodies (1, 2). In wAIHA, pre-transfusion screening for irregular antibodies will be positive, and crossmatching of type-identical blood will show incompatibility. Transfusion may hold a risk of acute or delayed hemolytic transfusion reaction and a risk of alloimmunization. When transfusion is clearly needed, however, this supportive therapy must not be withheld and is generally safe provided the required precautions are observed (154, 155). The old notion to use “the least incompatible donor blood” should be abandoned (156, 157). Instead, an extended phenotyping should be performed if time permits, and phenotype-identical erythrocyte concentrate should be preferred when possible (157, 158). A good communication between the clinician and the transfusion center is of vital importance.

In CAD, precautions are different. Transfusion is generally safer than in wAIHA and should not be omitted when indicated. However, there is a potential risk of agglutination and hemolysis of patient as well as donor erythrocytes because of cooling. Pre-transfusion screening and crossmatching will usually be unremarkable if performed at 37°C, but autoadsorption techniques may be required if the thermal amplitude of the cold agglutinin approaches 37°C (16). The patient and the extremity chosen for transfusion should be kept warm, and the use of an in-line blood warmer is recommended (1, 2, 20, 53).

## Conclusion

Prednisolone (or prednisone) at high initial doses remains the first-line treatment of wAIHA, but upfront addition of rituximab should be considered in severe cases. In CAD, effective therapies are directed at the pathogenic B-cell clone or the classical complement pathway. In both warm and cold-antibody mediated AIHA, the last decade has seen an increasing number of established and investigational treatment options, and individualization should be part of the therapeutic considerations. Above, we have outlined established or tentative advice for the choice of therapy in specific situations. In any type of AIHA, the ideal treatment remains to be developed, and some recommendations are still based on relatively weak evidence. Therefore, prospective clinical trials are important for future improvement of therapy in warm as well as cold-antibody mediated AIHA.

## Author contributions

SB collected data, prepared the tables and figures, and drafted the manuscript. BF and WB participated in data collection, and reviewed and commented the manuscript. All authors reviewed and approved the submitted version.
